# Supplementary material for: Interplay of BAF and MLL4 promotes cell type-specific enhancer activation
Source: Nat Commun. 2021 Mar 12;12:1630. doi: 10.1038/s41467-021-21893-y (PMC7955098; doi:10.1038/s41467-021-21893-y)
Supplement: Supplementary file 1 — Supplementary Information [file 41467_2021_21893_MOESM1_ESM.pdf]

Supplementary Information

Interplay of BAF and MLL4 promotes cell type-specific enhancer activation

Young-Kwon Park[1,#], Ji-Eun Lee[1,#], Zhijiang Yan[2,3], Kaitlin McKernan[1], Tommy O'Haren[1], Weidong Wang[2], Weiqun Peng[4], and Kai Ge[1,*]

[1]Adipocyte Biology and Gene Regulation Section, National Institute of Diabetes and Digestive and Kidney Diseases, National Institutes of Health (NIH), Bethesda, MD 20892
[2]Laboratory of Genetics and Genomics, National Institute on Aging, NIH, Baltimore, MD 21224
[3]School of Basic Medical Sciences, Wenzhou Medical University, Wenzhou 325035, China
[4]Department of Physics, The George Washington University, Washington, DC 20052
[#]These authors contributed equally

*To whom correspondence should be addressed. (Email: kai.ge@nih.gov)

Supplementary Figures 1-7
Supplementary Table 1
Supplementary References

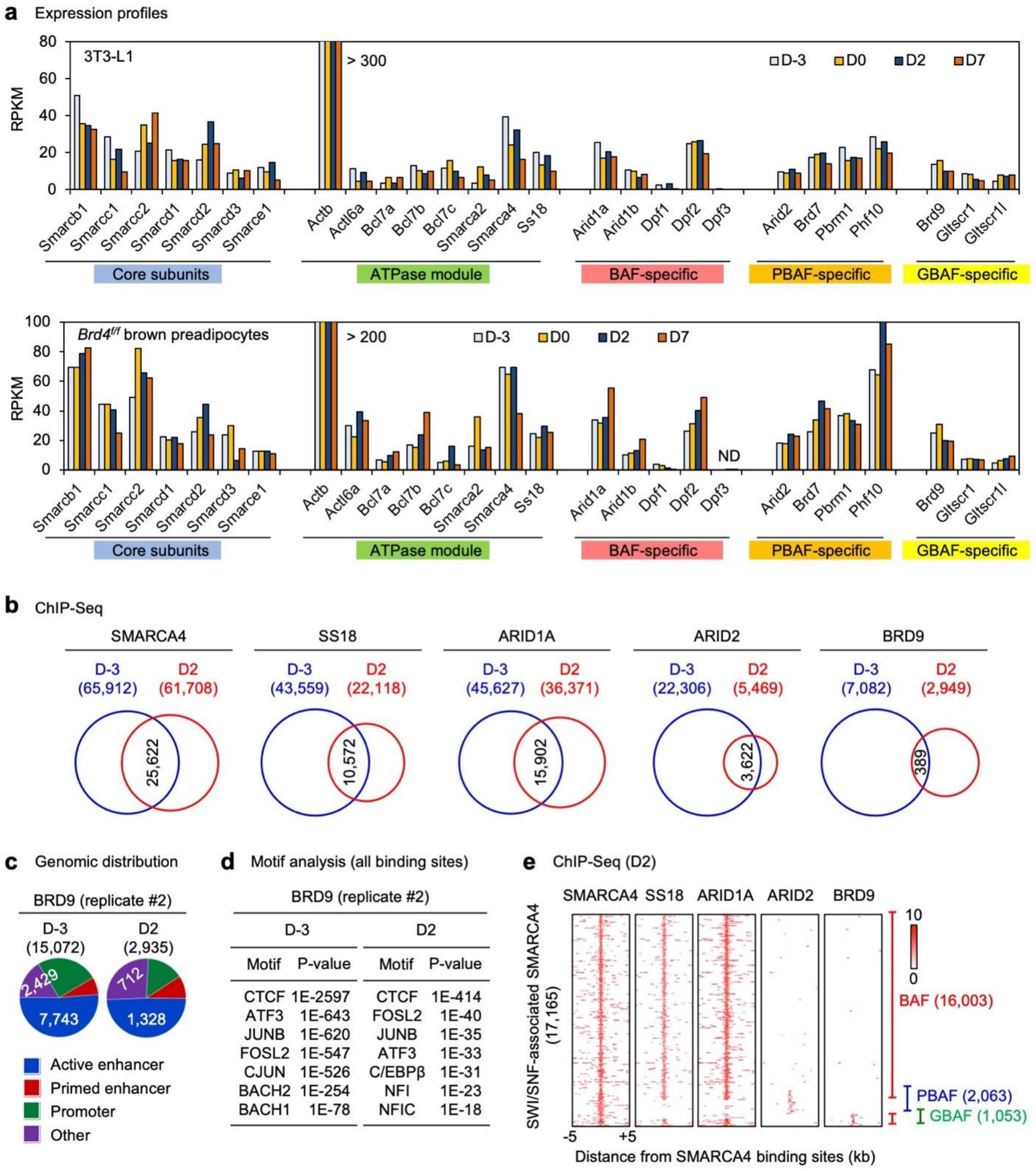

Supplementary Fig. 1. Genomic binding of SWI/SNF subunits in adipogenesis

(**a**) Expression profiles of SWI/SNF subunits during adipogenesis of 3T3-L1 white preadipocytes (n = 1) (GSE87113)[1] and *Brd4*$^{f/f}$ brown preadipocytes (n = 1) (GSE99101)[2]. D-3, day -3. D2, day 2.

(**b**) Venn diagrams depicting genomic binding of SMARCA4, SS18, BAF-specific ARID1A, PBAF-specific ARID2 and GBAF-specific BRD9 before (D-3) and during (D2) adipogenesis of brown preadipocytes. Numbers of binding sites are indicated.

(**c**) Genomic distributions of GBAF-specific BRD9 before (D-3) and during (D2) adipogenesis of brown preadipocytes. Confident BRD9 binding regions were determined by overlapping peaks from biological duplicates. Promoters were defined as transcription start sites ± 1kb. Active enhancers were defined as H3K4me1^{+} H3K27ac^{+} promoter-distal regions. Primed enhancers were defined as H3K4me1^{+} H3K27ac^{-} promoter-distal regions. Numbers of binding sites are indicated.
(**d**) Motif analysis of Brd9 binding regions at D-3 and D2 of adipogenesis using SeqPos motif tool. All binding regions were used.
(**e**) Heat maps for genomic binding of SMARCA4, SS18, ARID1A, ARID2 and BRD9 are shown around SMARCA4 binding sites at D2 of adipogenesis. Regions associated with either BAF, PBAF or GBAF are shown.

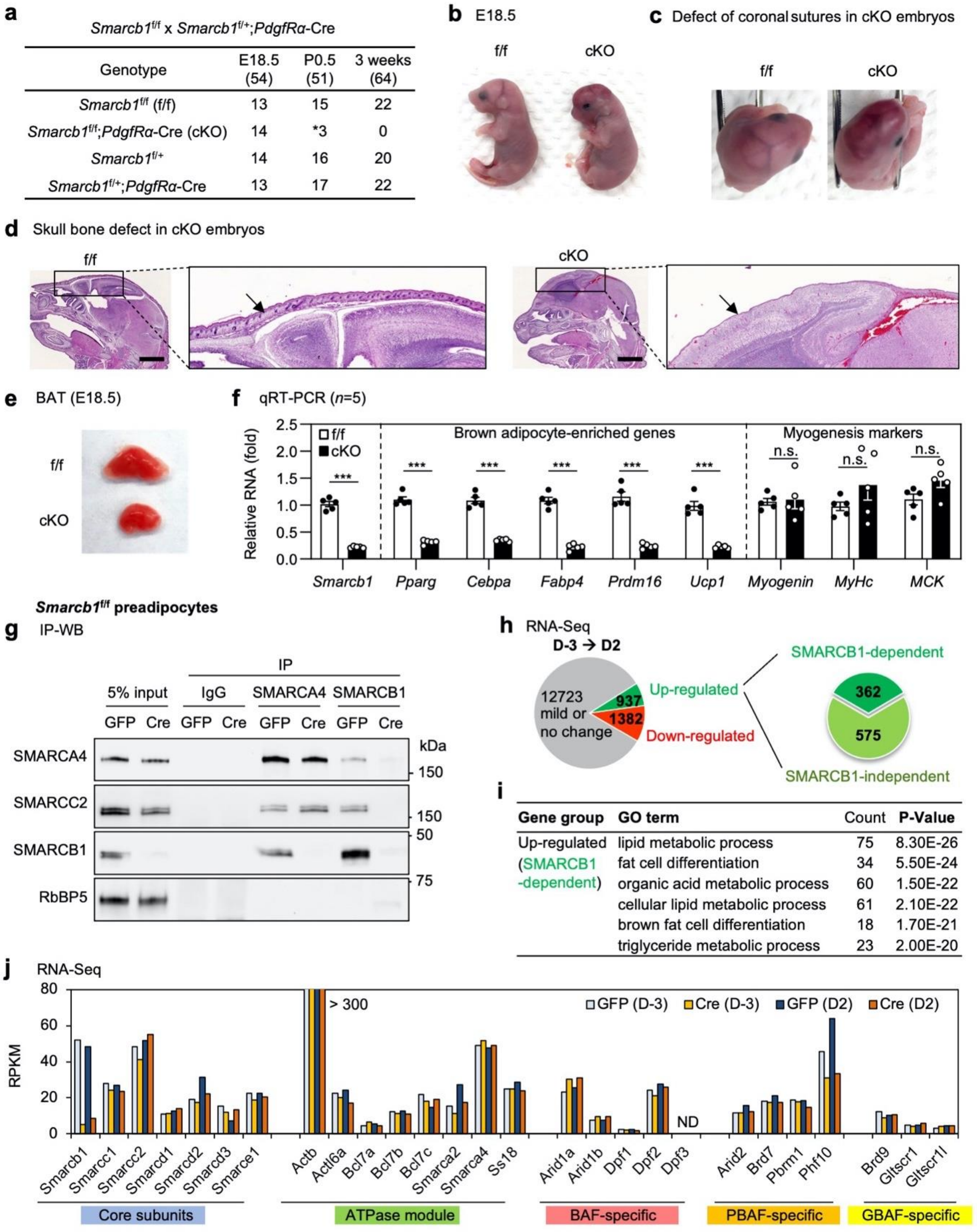

Supplementary Fig. 2. SMARCB1 is required for adipogenesis *in vivo* and in culture

(**a-d**) *Smarcb1*f/f;*PdgfRα-Cre* (cKO) mice showed defects in skull bone and died shortly after birth.

(**a**) Genotype of progeny from the crossing between *Smarcb1*$^{f/f}$ (f/f) and *Smarcb1*$^{f/+}$;*PdgfRα-Cre* mice at embryonic day 18.5 (E18.5), post-natal day 0.5 (P0.5) and weaning (3 weeks of age). Dead pups are indicated by an asterisk.

(**b**) Representative morphology of E18.5 embryos. (**c**) Defective coronal sutures in cKO mice.

(**d**) H&E staining of the sagittal sections of head. Arrows indicate the skull bone. *n* = 2 biological replicates. Scale bar = 1 mm.

(**e, f**) Impaired BAT development in cKO mice.

(**e**) Representative pictures of interscapular BAT of E18.5 f/f and cKO embryos.

(**f**) qRT-PCR analysis of *Smarcb1*, adipocyte-enriched genes *Pparg, Cebpa, Fabp4, Prdm16* and *Ucp1* as well as myogenesis marker genes *Myogenin, MyHC* and *MCK* in BAT isolated from E18.5 f/f and cKO embryos. Data are presented as mean values ± SEM. *n* = 5 independent experiments. Statistical comparison between groups was performed using the two-tailed unpaired *t*-test. *** p<0.001. n.s., not significant. Exact p-values are as follows: *Smarcb1* (p=1.9E-7); *Pparg* (p=4.1E-7); *Cebpa* (p=1.7E-6); *Fabp4* (p=1.1E-6); *Prdm16* (p=6.1E-6); *Ucp1* (p=1.6E-5); *Myogenin* (p=0.83); *MyHC* (p=0.21); *MCK* (p=0.06).

(**g-j**) SMARCB1 is required for adipogenesis in culture. Immortalized *Smarcb1*$^{f/f}$ brown preadipocytes were infected with adenoviral GFP or Cre, followed by adipogenesis assays. Cells were collected at D-3 and D2 of adipogenesis for RNA-Seq.

(**g**) Deletion of *Smarcb1* does not affect the interaction between SMARCA4 and SMARCC2. Nuclear extracts prepared at D2 of adipogenesis were immunoprecipitated (IP) with anti-SMARCA4 or anti-SMARCB1 antibody. Immunoprecipitates were analyzed by Western blot using antibodies indicated on the left. *n* = 2 biological replicates.

(**h**) RNA-Seq analysis at D2 of adipogenesis. Pie chart depicts SMARCB1-dependent and -independent up-regulated genes as well as down-regulated genes from D-3 to D2 of adipogenesis. The cut-off for differential expression is 2.5-fold.

(**i**) Gene ontology (GO) analysis of 362 SMARCB1-dependent up-regulated genes defined in (**h**) using DAVID software.

(**j**) Deletion of *Smarcb1* does not affect the expression of other SWI/SNF subunits in adipogenesis (*n* = 1).

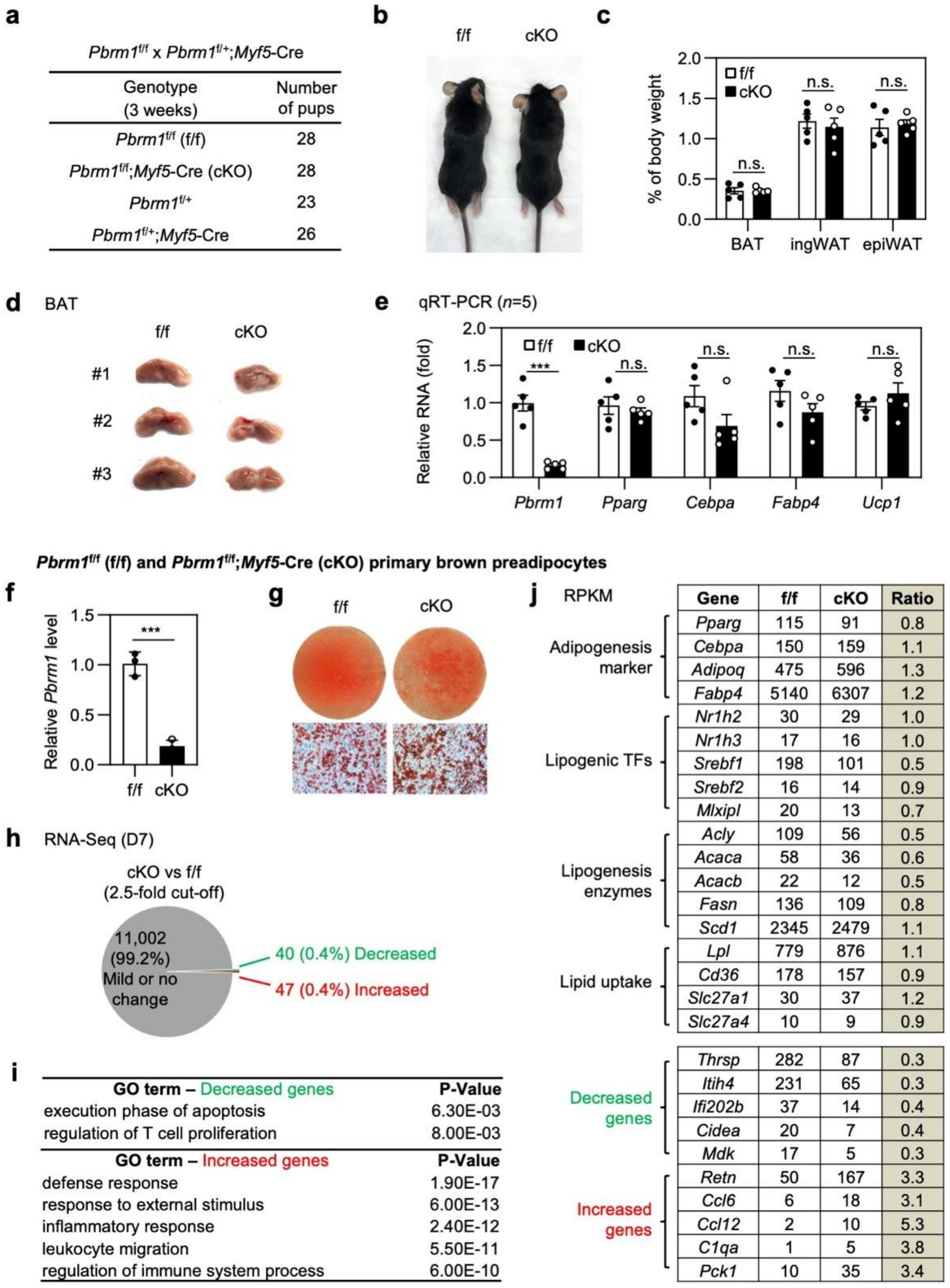

Supplementary Fig. 3. PBAF-specific subunit PBRM1 is dispensable for adipogenesis *in vivo* and in culture

(**a-e**) Characterization of adult (8 week-old) *Pbrm1*$^{f/f}$;*Myf5-Cre* (cKO) mice. *Pbrm1*$^{f/f}$ (f/f) were crossed with *Pbrm1*$^{f/+}$;*Myf5-Cre* to obtain cKO mice.

(**a**) Genotyping results. The expected ratios of the four genotypes are 1:1:1:1.

(**b**) Representative pictures of 8-week-old f/f or cKO mice.

(**c**) The average adipose tissue weights in f/f and cKO mice are shown as % of body weight. Data are presented as mean values ± SEM. *n* = 5 independent experiments. Statistical comparison between groups was performed using the two-tailed unpaired *t*-test. n.s., not significant.

(**d**) Pictures of interscapular BAT isolated from three f/f or cKO mice.

(**e**) Total RNA was extracted from BAT of f/f or cKO mice for qRT-PCR analysis of *Pbrm1*, adipogenesis markers *Pparg*, *Cebpα* and *Fabp4* and BAT marker *Ucp1*. Data are presented as mean values ± SEM. *n* = 5 independent experiments. Statistical comparison between groups was performed using the two-tailed unpaired *t*-test. *** p<0.001. n.s., not significant. Exact p-values are as follows: *Pbrm1* (p=5.3E-5); *Pparg* (p=0.53); *Cebpa* (p=0.09); *Fabp4* (p=0.15); *Ucp1* (p=0.30).

(**f**-**j**) Pbrm1 is dispensable for adipogenesis in cell culture. Primary brown preadipocytes were isolated from interscapular BAT of f/f or cKO mouse at P0.5, followed by adipogenesis until D7.

(**f**) *Pbrm1* deletion in primary brown preadipocytes was confirmed by qPCR of genomic DNA. Expression levels were normalized to *18S rRNA*. *n* = 3 biologically independent samples. Data are presented as mean values ± std. dev. *** p<0.001. Exact p-value for *Pbrm1* is p=4.2E-4.

(**g**) Oil Red O staining of differentiated cells at D7.

(**h**) RNA-Seq analysis of gene expression at D7 (*n* = 3 biologically independent samples). Pie chart depicts decreased or increased genes in cKO versus f/f cells. The cut-off is 2.5-fold.

(**i**) Gene ontology (GO) analysis of decreased or increased genes defined in (**H**).

(**j**) The list of representative adipocyte differentiation genes (upper panel) and significantly decreased or increased genes in *Pbrm1* cKO cells at D7 (lower panel). RPKM values indicate gene expression levels.

a Motif analysis: SMARCA4⁺ AE

D-3		D2	
Motif	P-value	Motif	P-value
JUN	7E-105	C/EBPβ	2E-87
JUNB	7E-103	C/EBPα	5E-87
FOSL1	6E-101	C/EBPδ	7E-77
FOS	2E-94	C/EBPγ	9E-77
JUND	2E-93	NFIL3	5E-37
BACH2	9E-86	HLF	7E-36
JDP2	2E-80	ATF4	2E-32
BACH1	2E-77	DBP	1E-28
NFE2L2	1E-44	NFIX	3E-25
MAFF	6E-18	NFIC	3E-24

b Motif analysis: SS18⁺ AE

D-3		D2	
Motif	P-value	Motif	P-value
JUN	3E-149	C/EBPα	2E-275
JUNB	4E-143	C/EBPβ	3E-246
FOS	1E-139	C/EBPδ	4E-214
FOSL1	2E-126	C/EBPγ	2E-188
JUND	6E-126	JUND	6E-81
JDP2	7E-115	NFIL3	5E-76
BACH1	2E-107	JUN	2E-75
BACH2	4E-97	NFIC	2E-69
MAF	4E-60	JUNB	5E-68
NFE2L2	2E-55	NFIA	6E-67

c Motif analysis: ARID1A⁺ AE

D-3		D2	
Motif	P-value	Motif	P-value
FOS	1E-53	C/EBPα	2E-71
JDP2	3E-53	C/EBPβ	1E-56
JUNB	3E-49	C/EBPδ	4E-48
FOSL1	1E-48	C/EBPγ	2E-43
JUND	2E-48	NFIL3	2E-23
BACH1	2E-48	ATF4	1E-18
JUN	2E-46	HLF	8E-18
BACH2	2E-39	DBP	2E-14
NFE2	6E-39	NFIX	3E-12
BATF	4E-21	EBF	4E-11

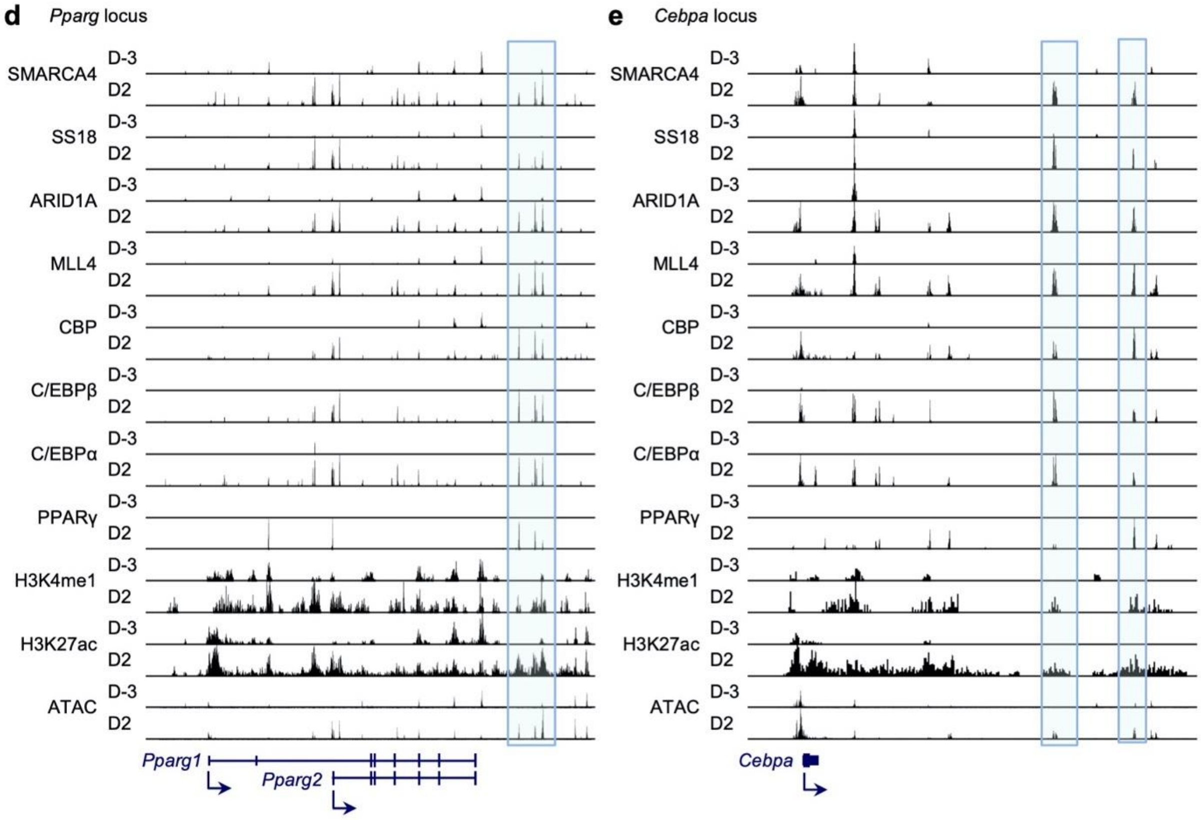

Supplementary Fig. 4. BAF co-localizes with LDTFs on active enhancers during adipogenesis

(**a-c**) Motif analysis of SMARCA4⁺ (**a**), SS18⁺ (**b**), or ARID1A⁺ (**c**) active enhancers (AEs) at D-3 and D2 of adipogenesis of brown preadipocytes. Top 3,000 binding regions on AEs were used for motif analysis. (**d-e**) Genome browser shot of ChIP-Seq data of BAF subunits (SMARCA4, SS18 and ARID1A), MLL4, CBP, LDTFs (C/EBPβ, C/EBPα, and PPARγ), H3K4me1, and H3K27ac as well as ATAC-Seq data on AEs of *Pparg* (**D**) or *Cebpa* (**E**) gene loci.

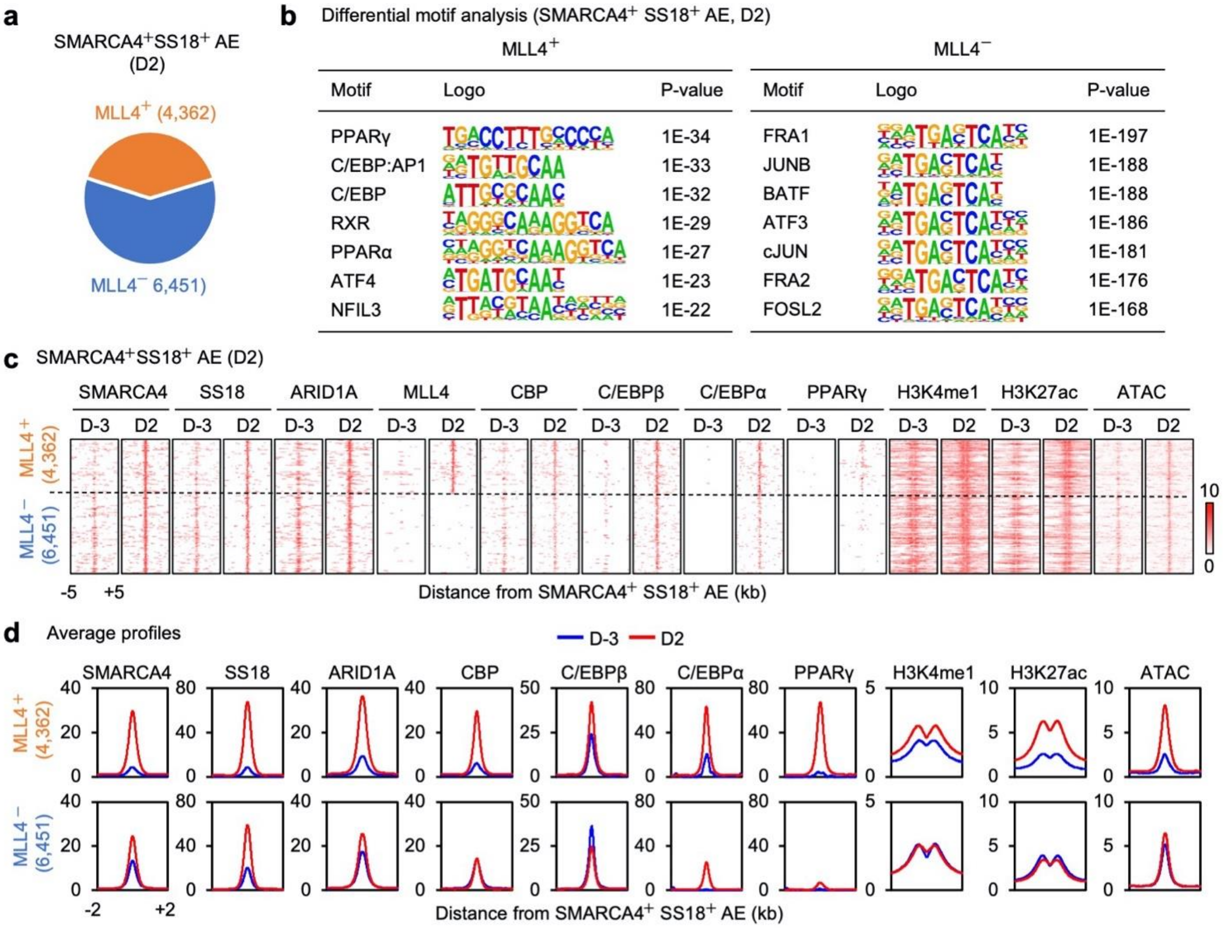

Supplementary Fig. 5. Markedly increased BAF binding and chromatin accessibility on MLL4⁺ active enhancers during adipogenesis

(**a**) Among the 10,813 BAF-binding (SMARCA4⁺ SS18⁺) active enhancers (AEs) at D2 of adipogenesis of brown preadipocytes, 4,362 are bound by MLL4.

(**b**) Differential motif analysis of MLL4⁺ or MLL4⁻ AEs at D2 of adipogenesis using HOMER.

(**c-d**) Heat maps (**c**) and average profiles (**d**) around SMARCA4⁺ SS18⁺ AEs. Levels of CBP and H3K27ac were only induced on MLL4⁺ AEs. Normalized read counts are shown.

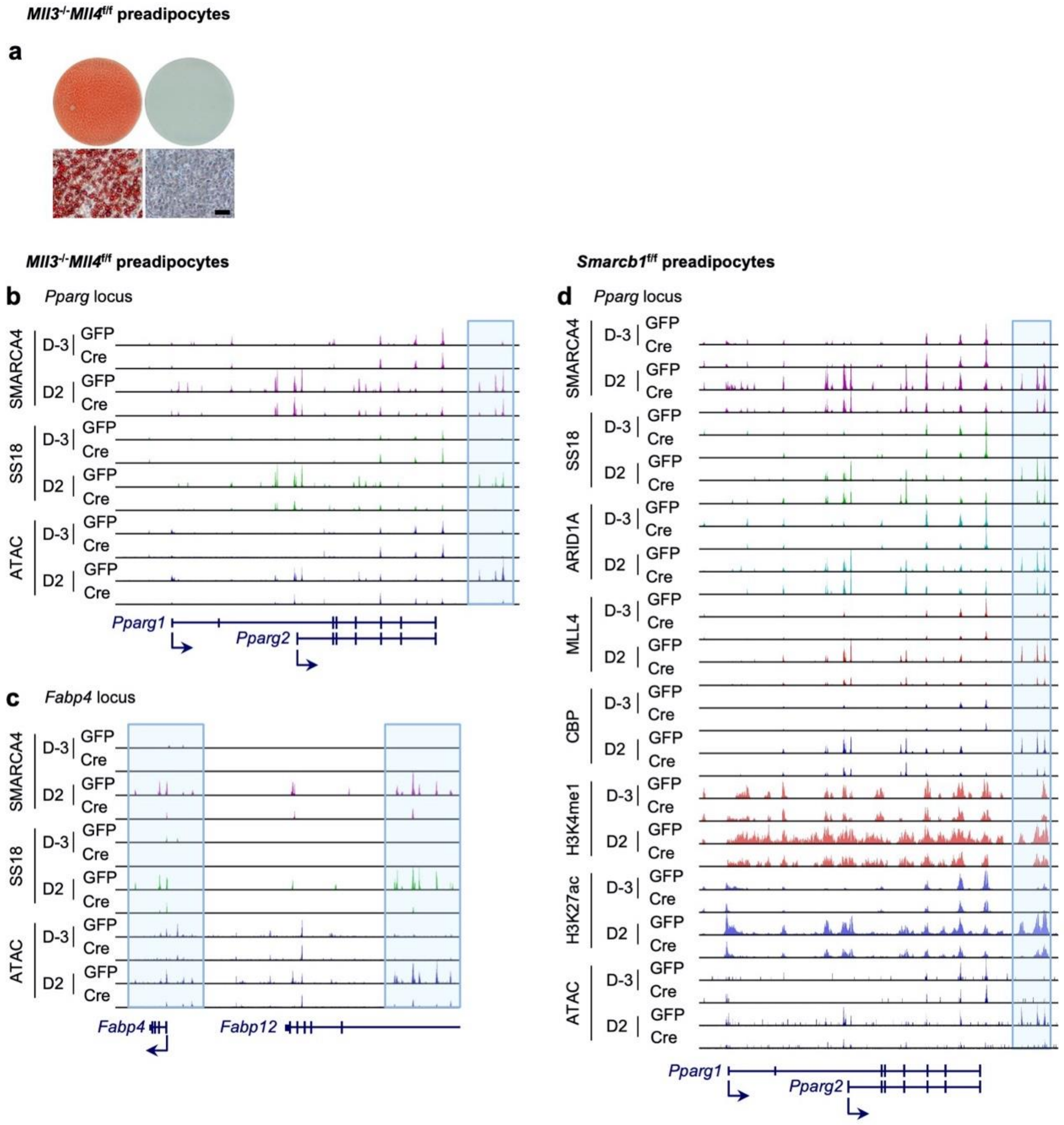

Supplementary Fig. 6. Reciprocal regulation between BAF and MLL4 on active enhancers during adipogenesis

(**a**) MLL4 is required for adipogenesis of brown preadipocytes. Oil Red O staining at D7. *n* = 3 biological replicates. Scale bar = 50 μm.

(**b, c**) MLL4 is required for SMARCA4 and SS18 binding and chromatin opening on adipogenic enhancers around *Pparg* (**b**) and *Fabp4* (**c**) genes at D2 of adipogenesis.

(**d**) SMARCB1 is required for SMARCA4, MLL4, and CBP binding and activation of enhancers around the *Pparg* gene at D2 of adipogenesis.

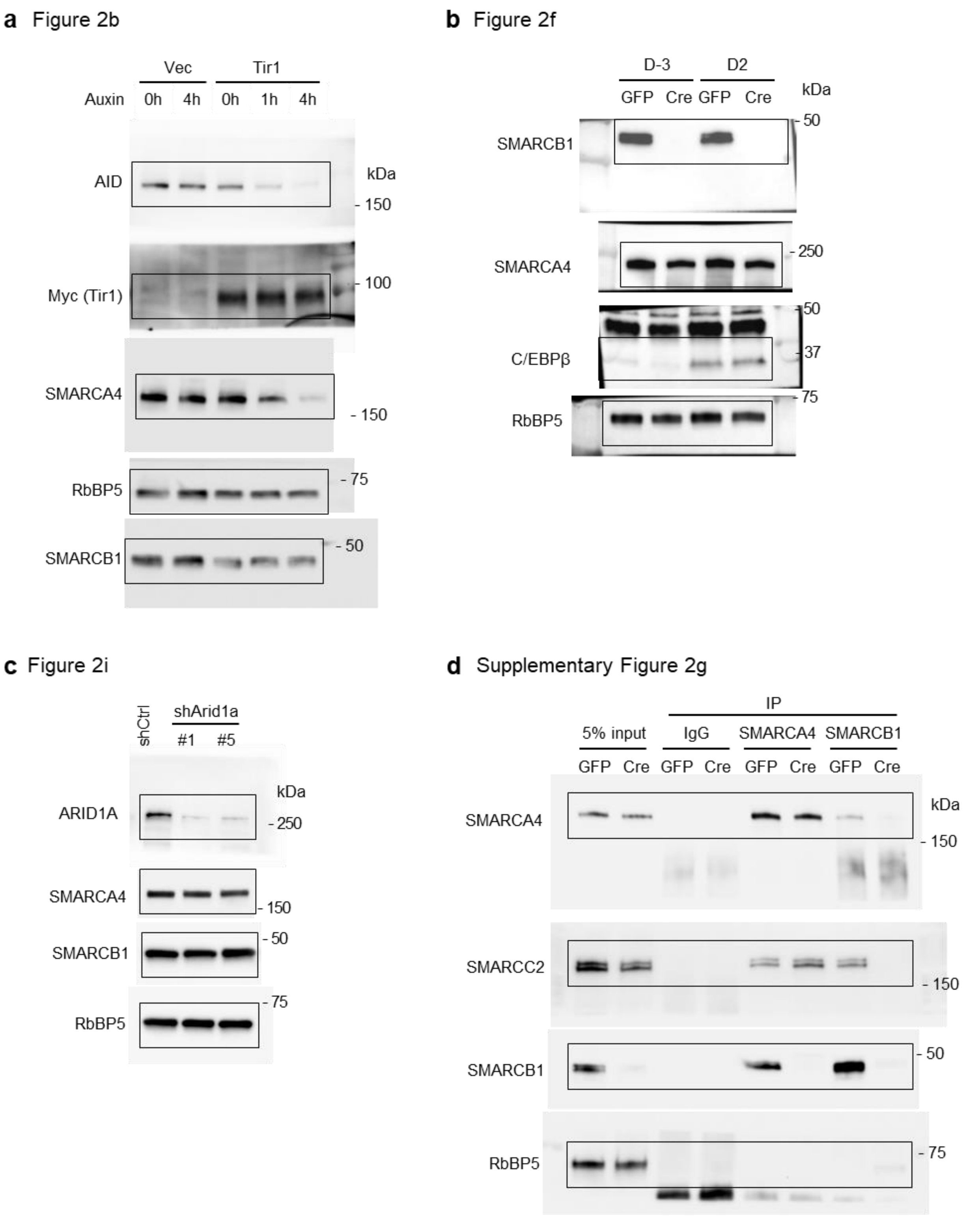

Supplementary Fig. 7. Uncropped Western blots.

Uncropped Western blots related to Figure 2b (**a**), Figure 2f (**b**), Figure 2i (**c**), and Supplementary Figure 2g (**d**).

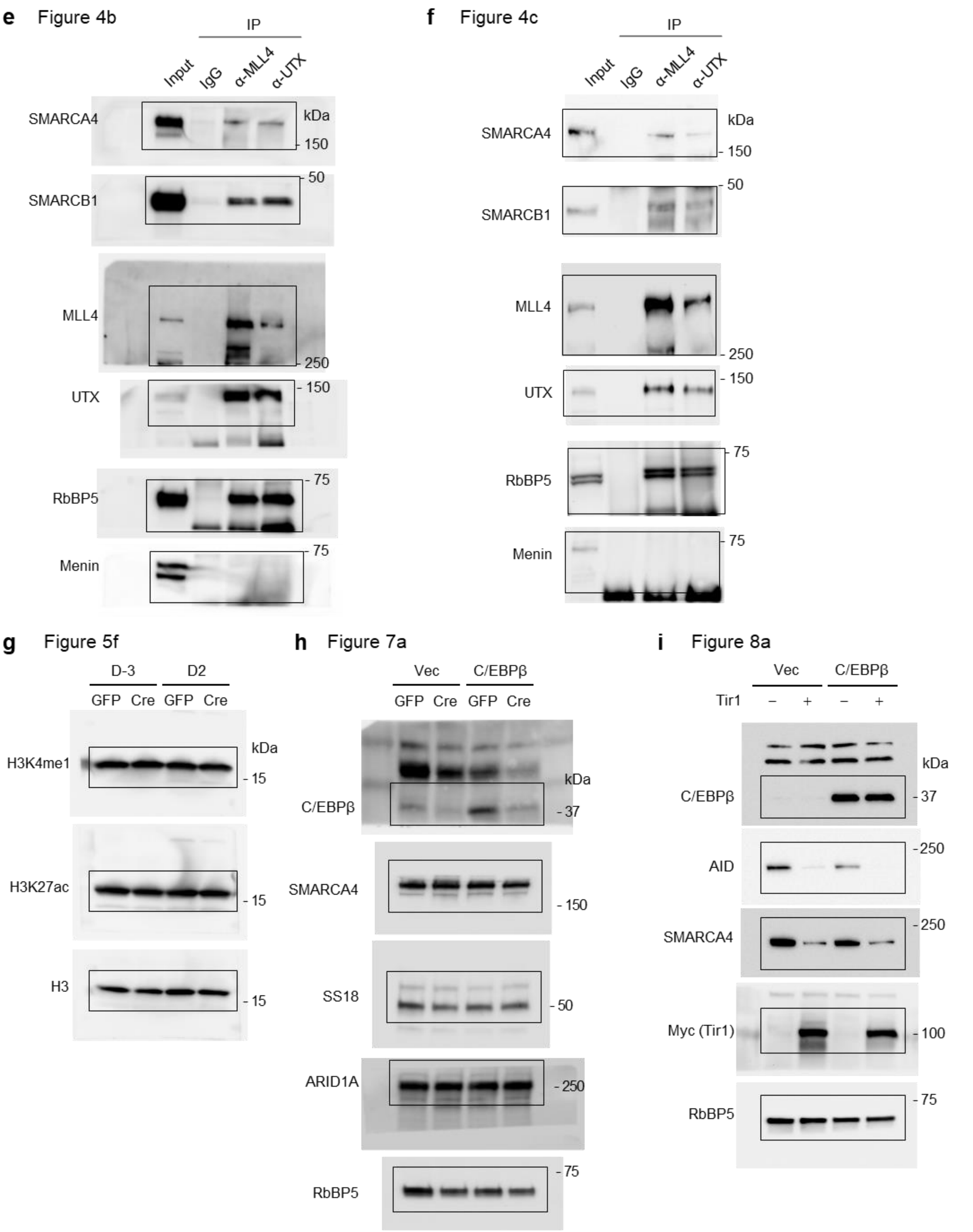

Supplementary Fig. 7. Uncropped Western blots (continue).

Uncropped Western blots related to Figure 4b (**e**), Figure 4c (**f**), Figure 5f (**g**), Figure 7a (**h**), and Figure 8a (**i**).

Supplementary Table 1. SYBR green primer sequences for qRT-PCR

Gene	Forward (5' → 3')	Reverse (5' → 3')
18s	ATGCCCTGCCCTTTGTACACA	CGATCCGAGGGCCTCACTA
Arid1a	TGGGCAAGATGAGACCTCAG	TCTGCTGTGCATAAGAGAGGC
Cebpa	CAAGAACAGCAACGAGTACCG	GTCACTGGTCAACTCCAGCAC
Cebpb	AAGCTGAGCGACGAGTACAAGA	GTCAGCTCCAGCACCTTGTG
Fabp4	GGGGCCAGGCTTCTATTCC	GGAGCTGGGTTAGGTATGGG
MCK	ACTACAAGCCTCAGGAGTA	TTATCGCGAAGCTTATTGTAG
MyHC	TTGAAAAGACGAAGCAGCGAC	AGAGAGCGGGACTCCTCCTG
Myogenin	AGGCTGGGTGTGCATGTGA	TTAAAAGCCCCCTGCTACAGAAG
Pbrm1	GAAAGAGCTGGGCCCTCTG	CAGTTTCTGCTGCATTGGAGT
Pparg	TCGCTGATGCACTGCCTATG	GAGAGGTCCACAGAGCTGATT
Prdm16	CAGCACGGTGAAGCCATTC	GCGTGCATCCGCTTGTG
Smarcb1	CACCATGCCCCACCTCCCCTACA	CAGGAAAATGGATGCAACTAAGAT
Ucp1	GGCCTCTACGACTCAGTCCA	TAAGCCGGCTGAGATCTTGT

Supplementary References

1 Park, Y. K. *et al.* Distinct Roles of Transcription Factors KLF4, Krox20, and Peroxisome Proliferator-Activated Receptor gamma in Adipogenesis. *Mol Cell Biol* **37**, doi:10.1128/MCB.00554-16 (2017).

2 Lee, J. E. *et al.* Brd4 binds to active enhancers to control cell identity gene induction in adipogenesis and myogenesis. *Nat Commun* **8**, 2217, doi:10.1038/s41467-017-02403-5 (2017).
