# Supplementary material for: Interplay of BAF and MLL4 promotes cell type-specific enhancer activation
Source: Nat Commun. 2021 Mar 12;12:1630. doi: 10.1038/s41467-021-21893-y (PMC7955098; doi:10.1038/s41467-021-21893-y)
Supplement: Supplementary file 3 — Reporting Summary [file 41467_2021_21893_MOESM3_ESM.pdf]

nature research

Corresponding author(s): Kai Ge

Last updated by author(s): Jan 8, 2021

Reporting Summary

Nature Research wishes to improve the reproducibility of the work that we publish. This form provides structure for consistency and transparency in reporting. For further information on Nature Research policies, see our Editorial Policies and the Editorial Policy Checklist.

Statistics

For all statistical analyses, confirm that the following items are present in the figure legend, table legend, main text, or Methods section.

n/a	Confirmed	
☐	☒	The exact sample size (*n*) for each experimental group/condition, given as a discrete number and unit of measurement
☐	☒	A statement on whether measurements were taken from distinct samples or whether the same sample was measured repeatedly
☐	☒	The statistical test(s) used AND whether they are one- or two-sided<br>*Only common tests should be described solely by name; describe more complex techniques in the Methods section.*
☒	☐	A description of all covariates tested
☒	☐	A description of any assumptions or corrections, such as tests of normality and adjustment for multiple comparisons
☐	☒	A full description of the statistical parameters including central tendency (e.g. means) or other basic estimates (e.g. regression coefficient) AND variation (e.g. standard deviation) or associated estimates of uncertainty (e.g. confidence intervals)
☐	☒	For null hypothesis testing, the test statistic (e.g. *F*, *t*, *r*) with confidence intervals, effect sizes, degrees of freedom and *P* value noted<br>*Give P values as exact values whenever suitable.*
☒	☐	For Bayesian analysis, information on the choice of priors and Markov chain Monte Carlo settings
☒	☐	For hierarchical and complex designs, identification of the appropriate level for tests and full reporting of outcomes
☒	☐	Estimates of effect sizes (e.g. Cohen's *d*, Pearson's *r*), indicating how they were calculated

Our web collection on statistics for biologists contains articles on many of the points above.

Software and code

Policy information about availability of computer code

Data collection	No software was used to collect data.
Data analysis	Software for RNA-Seq, ChIP-Seq and ATAC-Seq analysis include STAR(v.2.7.6), DAVID (v.6.7), bowtie2(v.2.3.4.1), SICER algorithm(v.1.1), SeqPos, Homer(v.4.10.4), and ATAC-Seataqc pipelines listed in the Methods section. SEQUEST (v.28) was used for peptide identification from IP-MS data.

For manuscripts utilizing custom algorithms or software that are central to the research but not yet described in published literature, software must be made available to editors and reviewers. We strongly encourage code deposition in a community repository (e.g. GitHub). See the Nature Research guidelines for submitting code & software for further information.

Data

Policy information about availability of data

All manuscripts must include a data availability statement. This statement should provide the following information, where applicable:

- Accession codes, unique identifiers, or web links for publicly available datasets
- A list of figures that have associated raw data
- A description of any restrictions on data availability

All ChIP-Seq, RNA-Seq and ATAC-Seq datasets described in this paper have been deposited in NCBI Gene Expression Omnibus under access #GSE151115 [https://www.ncbi.nlm.nih.gov/geo/query/acc.cgi?acc=GSE151115]. The raw mass spectrometry proteomics data are provided in Supplementary Data 1 and 2. No replicates and no statistical validation are provided for the mass spectrometry data. Protein sequence databases were downloaded from Uniprot [https://www.uniprot.org/]. All other source data are provided with this paper.

Field-specific reporting

Please select the one below that is the best fit for your research. If you are not sure, read the appropriate sections before making your selection.

☒ Life sciences ☐ Behavioural & social sciences ☐ Ecological, evolutionary & environmental sciences

For a reference copy of the document with all sections, see nature.com/documents/nr-reporting-summary-flat.pdf

Life sciences study design

All studies must disclose on these points even when the disclosure is negative.

Sample size	Sample size is present in relevant figure legends. No sample size calculation was performed. Animal sample size was determined based on our previous experimental approach.
Data exclusions	No data exclusions
Replication	Replication is present in relevant figure legends.
Randomization	Mice with the same genotypes were randomly chosen for different assays (H&E staining, gene expression study).
Blinding	Blinding was not necessary because measurements were empirical and not subjective.

Reporting for specific materials, systems and methods

We require information from authors about some types of materials, experimental systems and methods used in many studies. Here, indicate whether each material, system or method listed is relevant to your study. If you are not sure if a list item applies to your research, read the appropriate section before selecting a response.

Materials & experimental systems

n/a	Involved in the study	
☐	☒	Antibodies
☐	☒	Eukaryotic cell lines
☒	☐	Palaeontology and archaeology
☐	☒	Animals and other organisms
☒	☐	Human research participants
☒	☐	Clinical data
☒	☐	Dual use research of concern

Methods

n/a	Involved in the study	
☐	☒	ChIP-seq
☒	☐	Flow cytometry
☒	☐	MRI-based neuroimaging

Antibodies

Antibodies used	Anti-SMARCB1/SNF5/INI1 (A-5, sc-166165), anti-SMARCC2/BAF170 (E-6, sc-17838X), anti-ARID2/BAF200 (E-3, sc-166117X), anti-C/EBPβ (C-19, sc-150X), anti-C/EBPα (144AA, sc-61X) and anti-PPARγ (H-100, sc-7196X) were from Santa Cruz. Anti-RbBP5 (A300-109A) was from Bethyl Laboratories. Anti-SMARCA4/BRG1 (ab110641) and anti-H3K27ac (ab4729) were from Abcam. Anti-ARID1A/BAF250A (D2A8U, #12354), anti-SS18 (D6I4Z, #21792) and anti-CBP (D6C5, #7389) were from Cell Signaling. Anti-BRD9 (#61537) was from Active Motif. Anti-H3K4me1 (13-0040) was from EpiCypher. Homemade anti-UTX and anti-MLL4 were used. For fluorescent secondary antibodies, anti-mouse Alexa Fluor 488 and anti-rabbit Alexa Fluor 555 (Life Technologies, Carlsbad, CA, USA) were used.
Validation	Anti-SMARCB1/SNF5/INI1, Anti-SMARCA4/BRG1 (ab110641), anti-CBP (D6C5, #7389), homemade anti-UTX and anti-MLL4 were validated using knockout cells as negative controls. Anti-C/EBPβ (C-19, sc-150X), anti-C/EBPα (144AA, sc-61X), anti-PPARγ (H-100, sc-7196X), anti-RbBP5 (A300-109A), and anti-H3K27ac (ab4729) were validated in previous studies from our laboratory. Anti-SMARCC2/BAF170 (E-6, sc-17838X), anti-ARID1A/BAF250A (D2A8U, #12354), anti-SS18 (D6I4Z, #21792) were validated for detecting mouse proteins by WB in the manufacturers' website. Anti-H3K4me1 (13-0040) was validated by SNAP-ChIP-Seq by the manufacturer.

Eukaryotic cell lines

Policy information about cell lines

Cell line source(s)	Brown preadipocyte cell lines were generated from conditional knockout mice and described in the Methods section. HEK293T cell line was obtained from ATCC.
Authentication	None of the cell lines used were authenticated.

Mycoplasma contamination	Cell lines were not tested for mycoplasma contamination.
Commonly misidentified lines (See ICLAC register)	No commonly misidentified cell line was used in this study.

Animals and other organisms

Policy information about studies involving animals; ARRIVE guidelines recommended for reporting animal research

Laboratory animals	Smarcb1-flox/flox mice were obtained from Charles W.M. Roberts (St. Jude Children's Research Hospital, Memphis, TN), Pbrm1-flox/flox mice (Stock No. 029049), PdgfRα-Cre (Stock No.013148), Myf5-Cre (Stock No. 007893) mice were obtained from Jackson Laboratory. Smarca4-AID/AID mice were obtained from Keji Zhao (NHLBI, NIH, Bethesda, MD).
Wild animals	No wild animal was used in this research.
Field-collected samples	No field-collected samples were used.
Ethics oversight	All mouse work was approved by the Animal Care and Use Committee of NIDDK, NIH.

Note that full information on the approval of the study protocol must also be provided in the manuscript.

ChIP-seq

Data deposition

☒ Confirm that both raw and final processed data have been deposited in a public database such as GEO.

☒ Confirm that you have deposited or provided access to graph files (e.g. BED files) for the called peaks.

Data access links<br>*May remain private before publication.*	https://www.ncbi.nlm.nih.gov/geo/query/acc.cgi?acc=GSE151115
Files in database submission	BM34_GFP_D-3_BAF200.fastq.gz<br>BM34_GFP_D2_BAF200.fastq.gz<br>BM34_Cre_D-3_SS18.fastq.gz<br>BM34_Cre_D2_SS18.fastq.gz<br>BM34_GFP_D-3_SS18.fastq.gz<br>BM34_GFP_D2_SS18.fastq.gz<br>BMll34_Cre_D-3_Brg1_S41_L008_R1_001.fastq.gz<br>BMll34_Cre_D2_Brg1_S48_L008_R1_001.fastq.gz<br>BMll34_GFP_D-3_Brg1_S45_L008_R1_001.fastq.gz<br>BMll34_GFP_D2_Brg1_S40_L008_R1_001.fastq.gz<br>BM34_Cre_D-3_input.fastq.gz<br>BM34_Cre_D2_input.fastq.gz<br>BM34_GFP_D-3_input.fastq.gz<br>BM34_GFP_D2_input.fastq.gz<br>BMll34_Cre_D-3_ATAC.fastq.gz<br>BMll34_Cre_D2_ATAC_real.fastq.gz<br>BMll34_GFP_D-3_ATAC.fastq.gz<br>BMll34_GFP_D2_ATAC_real.fastq.gz<br>BSnf5_D2_CEBPa.fastq.gz<br>BSnf5_D2_CEBPb.fastq.gz<br>BSnf5_D2_PPARg.fastq.gz<br>BSnf5_Cre_D-3_Brg1.fastq.gz<br>BSnf5_Cre_D2_Brg1.fastq.gz<br>BSnf5_GFP_D-3_Brg1.fastq.gz<br>BSnf5_GFP_D2_Brg1.fastq.gz<br>BSnf5_Cre_D-3_Mll4.fastq.gz<br>BSnf5_Cre_D2_Mll4.fastq.gz<br>BSnf5_GFP_D-3_Mll4.fastq.gz<br>BSnf5_GFP_D2_Mll4.fastq.gz<br>BSnf5_Cre_D-3_CBP.fastq.gz<br>BSnf5_Cre_D2_CBP.fastq.gz<br>BSnf5_GFP_D-3_CBP.fastq.gz<br>BSnf5_GFP_D2_CBP.fastq.gz<br>BSnf5_Cre_D-3_H3K4me1.fastq.gz<br>BSnf5_Cre_D2_H3K4me1.fastq.gz<br>BSnf5_GFP_D-3_H3K4me1.fastq.gz<br>BSnf5_GFP_D2_H3K4me1.fastq.gz<br>BSnf5_Cre_D-3_H3K27ac.fastq.gz<br>BSnf5_Cre_D2_H3K27ac.fastq.gz<br>BSnf5_GFP_D-3_H3K27ac.fastq.gz<br>BSnf5_GFP_D2_H3K27ac.fastq.gz

BSnf5_GFP_D-3_Brd9.fastq.gz
BSnf5_GFP_D2_Brd9.fastq.gz
BSnf5_Cre_D-3_Arid1a.fastq.gz
BSnf5_Cre_D2_Arid1a.fastq.gz
BSnf5_GFP_D-3_Arid1a.fastq.gz
BSnf5_GFP_D2_Arid1a.fastq.gz
BSnf5_Cre_D-3_SS18.fastq.gz
BSnf5_Cre_D2_SS18.fastq.gz
BSnf5_GFP_D-3_SS18.fastq.gz
BSnf5_GFP_D2_SS18.fastq.gz
BSnf5_Cre_D-3_input.fastq.gz
BSnf5_Cre_D2_input.fastq.gz
BSnf5_GFP_D-3_input.fastq.gz
BSnf5_GFP_D2_input.fastq.gz
Snf5_Cre_D-3_RNA_S8_L002_R1_001.fastq.gz
Snf5_Cre_D2_RNA_S17_L002_R1_001.fastq.gz
Snf5_GFP_D-3_RNA_S16_L002_R1_001.fastq.gz
Snf5_GFP_D2_RNA_S13_L002_R1_001.fastq.gz
BSnf5_ATAC_Cre_D-3.fastq.gz
BSnf5_ATAC_Cre_D2.fastq.gz
BSnf5_ATAC_GFP_D-3.fastq.gz
BSnf5_ATAC_GFP_D2.fastq.gz
BMll34_Cebpb_Cre_D-3_Brg1.fastq.gz
BMll34_Cebpb_GFP_D-3_Brg1.fastq.gz
BMll34_Vec_Cre_D-3_Brg1.fastq.gz
BMll34_Vec_GFP_D-3_Brg1.fastq.gz
BMll34_Cebpb_Cre_D-3_input.fastq.gz
BMll34_Cebpb_GFP_D-3_input.fastq.gz
BMll34_Vec_Cre_D-3_input.fastq.gz
BMll34_Vec_GFP_D-3_input.fastq.gz
BMll34_Cebpb_Cre_D-3_Arid1a.fastq.gz
BMll34_Cebpb_GFP_D-3_Arid1a.fastq.gz
BMll34_Vec_Cre_D-3_Arid1a.fastq.gz
BMll34_Vec_GFP_D-3_Arid1a.fastq.gz
BMll34_Cebpb_Cre_D-3_ATAC.fastq.gz
BMll34_Cebpb_GFP_D-3_ATAC.fastq.gz
BMll34_Vec_Cre_D-3_ATAC.fastq.gz
BMll34_Vec_GFP_D-3_ATAC.fastq.gz
BPbrm1_Myf5Cre_RNA_D7
BPbrm1_RNA_flox_D7.fastq.gz
Brg1-AID_Cebpb_Tir1_D-3_ATAC.fastq.gz
Brg1-AID_Cebpb_Vec_D-3_ATAC.fastq.gz
Brg1-AID_Vec_Tir1_D-3_ATAC.fastq.gz
Brg1-AID_Vec_Vec_D-3_ATAC.fastq.gz
Brg1-AID_Cebpb_Tir1_D-3_Cebpb.fastq.gz
Brg1-AID_Cebpb_Vec_D-3_Cebpb.fastq.gz
Brg1-AID_Vec_Tir1_D-3_Cebpb.fastq.gz
Brg1-AID_Vec_Vec_D-3_Cebpb.fastq.gz
Brg1-AID_Cebpb_Tir1_D-3_H3K27ac.fastq.gz
Brg1-AID_Cebpb_Vec_D-3_H3K27ac.fastq.gz
Brg1-AID_Vec_Tir1_D-3_H3K27ac.fastq.gz
Brg1-AID_Vec_Vec_D-3_H3K27ac.fastq.gz
Brg1-AID_Cebpb_Tir1_D-3_input.fastq.gz
Brg1-AID_Cebpb_Vec_D-3_input.fastq.gz
Brg1-AID_Vec_Tir1_D-3_input.fastq.gz
Brg1-AID_Vec_Vec_D-3_input.fastq.gz
Brg1-AID_Cebpb_Tir1_D-3_Mll4.fastq.gz
Brg1-AID_Cebpb_Vec_D-3_Mll4.fastq.gz
Brg1-AID_Vec_Tir1_D-3_Mll4.fastq.gz
Brg1-AID_Vec_Vec_D-3_Mll4.fastq.gz
BM34_GFP_D-3_BAF200_sorted-W50-G50-FDR1E-3-islandfiltered-normalized.wig
BM34_GFP_D2_BAF200_sorted-W50-G50-FDR1E-3-islandfiltered-normalized.wig
BM34_Cre_D-3_SS18_sorted-W50-G50-FDR1E-10-islandfiltered-normalized.wig
BM34_Cre_D2_SS18_sorted-W50-G50-FDR1E-10-islandfiltered-normalized.wig
BM34_GFP_D-3_SS18_sorted-W50-G50-FDR1E-10-islandfiltered-normalized.wig
BM34_GFP_D2_SS18_sorted-W50-G50-FDR1E-10-islandfiltered-normalized.wig
Sample_BMll34_Cre_D-3_Brg1_sorted-W50-G50-FDR1e-10-islandfiltered-normalized.wig
Sample_BMll34_Cre_D2_Brg1_sorted-W50-G50-FDR1e-10-islandfiltered-normalized.wig
Sample_BMll34_GFP_D-3_Brg1_sorted-W50-G50-FDR1e-10-islandfiltered-normalized.wig
Sample_BMll34_GFP_D2_Brg1_sorted-W50-G50-FDR1e-10-islandfiltered-normalized.wig
BM34_Cre_D-3_input_sorted-W50-normalized.wig
BM34_Cre_D2_input_sorted-W50-normalized.wig
BM34_GFP_D-3_input_sorted-W50-normalized.wig
BM34_GFP_D2_input_sorted-W50-normalized.wig
BMll34_Cre_D-3_ATAC.nodup.tn5.pf.pval.signal.bigwig
BMll34_Cre_D2_ATAC_real.nodup.tn5.pf.pval.signal.bigwig

BMll34_GFP_D-3_ATAC.nodup.tn5.pf.pval.signal.bigwig
BMll34_GFP_D2_ATAC_real.nodup.tn5.pf.pval.signal.bigwig
BSnf5_D2_CEBPa_sorted-W50-G50-FDR1E-10-islandfiltered-normalized.wig
BSnf5_D2_CEBPb_sorted-W50-G50-FDR1E-10-islandfiltered-normalized.wig
BSnf5_D2_PPARg_sorted-W50-G50-FDR1E-10-islandfiltered-normalized.wig
BSnf5_Cre_D-3_Brg1_sorted-W50-G50-FDR1e-10-islandfiltered-normalized.wig
BSnf5_Cre_D2_Brg1_sorted-W50-G50-FDR1e-10-islandfiltered-normalized.wig
BSnf5_GFP_D-3_Brg1_sorted-W50-G50-FDR1e-10-islandfiltered-normalized.wig
BSnf5_GFP_D2_Brg1_sorted-W50-G50-FDR1e-10-islandfiltered-normalized.wig
BSnf5_Cre_D-3_Mll4_sorted-W50-G50-FDR1e-10-islandfiltered-normalized.wig
BSnf5_Cre_D2_Mll4_sorted-W50-G50-FDR1e-10-islandfiltered-normalized.wig
BSnf5_GFP_D-3_Mll4_sorted-W50-G50-FDR1e-10-islandfiltered-normalized.wig
BSnf5_GFP_D2_Mll4_sorted-W50-G50-FDR1e-10-islandfiltered-normalized.wig
BSnf5_Cre_D-3_CBP_sorted-W50-G50-FDR1e-10-islandfiltered-normalized.wig
BSnf5_Cre_D2_CBP_sorted-W50-G50-FDR1e-10-islandfiltered-normalized.wig
BSnf5_GFP_D-3_CBP_sorted-W50-G50-FDR1e-10-islandfiltered-normalized.wig
BSnf5_GFP_D2_CBP_sorted-W50-G50-FDR1e-10-islandfiltered-normalized.wig
BSnf5_Cre_D-3_H3K4me1_sorted-W200-G200-FDR1e-3-islandfiltered-normalized.wig
BSnf5_Cre_D2_H3K4me1_sorted-W200-G200-FDR1e-3-islandfiltered-normalized.wig
BSnf5_GFP_D-3_H3K4me1_sorted-W200-G200-FDR1e-3-islandfiltered-normalized.wig
BSnf5_GFP_D2_H3K4me1_sorted-W200-G200-FDR1e-3-islandfiltered-normalized.wig
BSnf5_Cre_D-3_H3K27ac_sorted-W200-G200-FDR1e-3-islandfiltered-normalized.wig
BSnf5_Cre_D2_H3K27ac_sorted-W200-G200-FDR1e-3-islandfiltered-normalized.wig
BSnf5_GFP_D-3_H3K27ac_sorted-W200-G200-FDR1e-3-islandfiltered-normalized.wig
BSnf5_GFP_D2_H3K27ac_sorted-W200-G200-FDR1e-3-islandfiltered-normalized.wig
BSnf5_GFP_D-3_Brd9_sorted-W50-G50-FDR1e-3-islandfiltered-normalized.wig
BSnf5_GFP_D2_Brd9_sorted-W50-G50-FDR1e-3-islandfiltered-normalized.wig
BSnf5_Cre_D-3_Arid1a_sorted-W50-G50-FDR1e-10-islandfiltered-normalized.wig
BSnf5_Cre_D2_Arid1a_sorted-W50-G50-FDR1e-10-islandfiltered-normalized.wig
BSnf5_GFP_D-3_Arid1a_sorted-W50-G50-FDR1e-10-islandfiltered-normalized.wig
BSnf5_GFP_D2_Arid1a_sorted-W50-G50-FDR1e-10-islandfiltered-normalized.wig
BSnf5_Cre_D-3_SS18_sorted-W50-G50-FDR1e-10-islandfiltered-normalized.wig
BSnf5_Cre_D2_SS18_sorted-W50-G50-FDR1e-10-islandfiltered-normalized.wig
BSnf5_GFP_D-3_SS18_sorted-W50-G50-FDR1e-10-islandfiltered-normalized.wig
BSnf5_GFP_D2_SS18_sorted-W50-G50-FDR1e-10-islandfiltered-normalized.wig
BSnf5_Cre_D-3_input_sorted-W50-normalized.wig
BSnf5_Cre_D2_input_sorted-W50-normalized.wig
BSnf5_GFP_D-3_input_sorted-W50-normalized.wig
BSnf5_GFP_D2_input_sorted-W50-normalized.wig
Sample_Snf5_Cre_D-3_RNA_sorted_on_Exon.txt
Sample_Snf5_Cre_D2_RNA_sorted_on_Exon.txt
Sample_Snf5_GFP_D-3_RNA_sorted_on_Exon.txt
Sample_Snf5_GFP_D2_RNA_sorted_on_Exon.txt
BSnf5_ATAC_Cre_D-3.nodup.tn5.pf.pval.signal.bigwig
BSnf5_ATAC_Cre_D2.nodup.tn5.pf.pval.signal.bigwig
BSnf5_ATAC_GFP_D-3.nodup.tn5.pf.pval.signal.bigwig
BSnf5_ATAC_GFP_D2.nodup.tn5.pf.pval.signal.bigwig
BMll34_Cebpb_Cre_D-3_Brg1_sorted-W50-G50-FDR1e-10-islandfiltered-normalized.wig
BMll34_Cebpb_GFP_D-3_Brg1_sorted-W50-G50-FDR1e-10-islandfiltered-normalized.wig
BMll34_Vec_Cre_D-3_Brg1_sorted-W50-G50-FDR1e-10-islandfiltered-normalized.wig
BMll34_Vec_GFP_D-3_Brg1_sorted-W50-G50-FDR1e-10-islandfiltered-normalized.wig
BMll34_Cebpb_Cre_D-3_input_sorted-W50-normalized.wig
BMll34_Cebpb_GFP_D-3_input_sorted-W50-normalized.wig
BMll34_Vec_Cre_D-3_input_sorted-W50-normalized.wig
BMll34_Vec_GFP_D-3_input_sorted-W50-normalized.wig
BMll34_Cebpb_Cre_D-3_Arid1a_sorted-W50-G50-FDR1E-10-islandfiltered-normalized.wig
BMll34_Cebpb_GFP_D-3_Arid1a_sorted-W50-G50-FDR1E-10-islandfiltered-normalized.wig
BMll34_Vec_Cre_D-3_Arid1a_sorted-W50-G50-FDR1E-10-islandfiltered-normalized.wig
BMll34_Vec_GFP_D-3_Arid1a_sorted-W50-G50-FDR1E-10-islandfiltered-normalized.wig
BMll34_Cebpb_Cre_D-3_ATAC.nodup.tn5.pf.pval.signal.bigwig
BMll34_Cebpb_GFP_D-3_ATAC.nodup.tn5.pf.pval.signal.bigwig
BMll34_Vec_Cre_D-3_ATAC.nodup.tn5.pf.pval.signal.bigwig
BMll34_Vec_GFP_D-3_ATAC.nodup.tn5.pf.pval.signal.bigwig
BPbrm1_RNA_Myf5Cre_D7_STAR_on_exon.txt.txt
BPbrm1_RNA_flox_D7_STAR_on_exon.txt.txt
Brg1-AID_Cebpb_Tir1_D-3_ATAC.nodup.tn5.pf.pval.signal.bigwig
Brg1-AID_Cebpb_Vec_D-3_ATAC.nodup.tn5.pf.pval.signal.bigwig
Brg1-AID_Vec_Tir1_D-3_ATAC.nodup.tn5.pf.pval.signal.bigwig
Brg1-AID_Vec_Vec_D-3_ATAC.nodup.tn5.pf.pval.signal.bigwig
Brg1-AID_Cebpb_Tir1_D-3_Cebpb_sorted-W50-G50-FDR1e-10-islandfiltered-normalized.wig
Brg1-AID_Cebpb_Vec_D-3_Cebpb_sorted-W50-G50-FDR1e-10-islandfiltered-normalized.wig
Brg1-AID_Vec_Tir1_D-3_Cebpb_sorted-W50-G50-FDR1e-10-islandfiltered-normalized.wig
Brg1-AID_Vec_Vec_D-3_Cebpb_sorted-W50-G50-FDR1e-10-islandfiltered-normalized.wig
Brg1-AID_Cebpb_Tir1_D-3_H3K27ac_sorted-W200-G200-FDR1e-3-islandfiltered-normalized.wig
Brg1-AID_Cebpb_Vec_D-3_H3K27ac_sorted-W200-G200-FDR1e-3-islandfiltered-normalized.wig
Brg1-AID_Vec_Tir1_D-3_H3K27ac_sorted-W200-G200-FDR1e-3-islandfiltered-normalized.wig

Brg1-AID_Vec_Vec_D-3_H3K27ac_sorted-W200-G200-FDR1e-3-islandfiltered-normalized.wig
Brg1-AID_Cebpb_Tir1_D-3_input_sorted-W50-normalized.wig
Brg1-AID_Cebpb_Vec_D-3_input_sorted-W50-normalized.wig
Brg1-AID_Vec_Tir1_D-3_input_sorted-W50-normalized.wig
Brg1-AID_Vec_Vec_D-3_input_sorted-W50-normalized.wig
Brg1-AID_Cebpb_Tir1_D-3_Mll4_sorted-W50-G50-FDR1e-3-islandfiltered-normalized.wig
Brg1-AID_Cebpb_Vec_D-3_Mll4_sorted-W50-G50-FDR1e-3-islandfiltered-normalized.wig
Brg1-AID_Vec_Tir1_D-3_Mll4_sorted-W50-G50-FDR1e-3-islandfiltered-normalized.wig
Brg1-AID_Vec_Vec_D-3_Mll4_sorted-W50-G50-FDR1e-3-islandfiltered-normalized.wig

Genome browser session
(e.g. UCSC)

no longer applicable

Methodology

Replicates

All RNA-Seq data are from single experiment. Most ChIP-Seq and ATAC-Seq data are from one representative of 2-3 highly reproducible biological replicates.

Sequencing depth

Mll3-/-Mll4f/f_BAF200_GFP_D-3 (17,291,395)
Mll3-/-Mll4f/f_BAF200_GFP_D2 (22,747,199)
Mll3-/-Mll4f/f_SS18_Cre_D-3 (27,001,862)
Mll3-/-Mll4f/f_SS18_Cre_D2 (20,564,576)
Mll3-/-Mll4f/f_SS18_GFP_D-3 (23,233,219)
Mll3-/-Mll4f/f_SS18_GFP_D2 (22,562,240)
Mll3-/-Mll4f/f_Brg1_Cre_D-3 (18,350,584)
Mll3-/-Mll4f/f_Brg1_Cre_D2 (19,683,010)
Mll3-/-Mll4f/f_Brg1_GFP_D-3 (16,261,636)
Mll3-/-Mll4f/f_Brg1_GFP_D2 (17,173,656)
Mll3-/-Mll4f/f_input_Cre_D-3 (28,540,266)
Mll3-/-Mll4f/f_input_Cre_D2 (22,031,624)
Mll3-/-Mll4f/f_input_GFP_D-3 (23,414,469)
Mll3-/-Mll4f/f_input_GFP_D2 (22,371,760)
Mll3-/-Mll4f/f_ATAC_Cre_D-3 (42,177,682)
Mll3-/-Mll4f/f_ATAC_Cre_D2 (64,374,234)
Mll3-/-Mll4f/f_ATAC_GFP_D-3 (36,349,422)
Mll3-/-Mll4f/f_ATAC_GFP_D2 (40,711,092)
Snf5f/f_CEBPa_GFP_D2 (47,536,975)
Snf5f/f_CEBPb_GFP_D2 (44,017,826)
Snf5f/f_PPARg_GFP_D2 (44,242,424)
Snf5f/f_Brg1_Cre_D-3 (33,142,374)
Snf5f/f_Brg1_Cre_D2 (27,791,949)
Snf5f/f_Brg1_GFP_D-3 (36,324,975)
Snf5f/f_Brg1_GFP_D2 (21,349,860)
Snf5f/f_MLL4_Cre_D-3 (35,756,917)
Snf5f/f_MLL4_Cre_D2 (32,250,379)
Snf5f/f_MLL4_GFP_D-3 (44,177,632)
Snf5f/f_MLL4_GFP_D2 (36,752,424)
Snf5f/f_CBP_Cre_D-3 (30,375,729)
Snf5f/f_CBP_Cre_D2 (25,549,128)
Snf5f/f_CBP_GFP_D-3 (26,223,273)
Snf5f/f_CBP_GFP_D2 (27,481,685)
Snf5f/f_H3K4me1_Cre_D-3 (32,426,333)
Snf5f/f_H3K4me1_Cre_D2 (31,181,753)
Snf5f/f_H3K4me1_GFP_D-3 (31,840,337)
Snf5f/f_H3K4me1_GFP_D2 (36,832,641)
Snf5f/f_H3K27ac_Cre_D-3 (33,960,513)
Snf5f/f_H3K27ac_Cre_D2 (31,262,497)
Snf5f/f_H3K27ac_GFP_D-3 (36,687,538)
Snf5f/f_H3K27ac_GFP_D2 (38,050,613)
Snf5f/f_Brd9_GFP_D-3 (18,196,007)
Snf5f/f_Brd9_GFP_D2 (31,217,010)
Snf5f/f_Arid1a_Cre_D-3 (24,986,064)
Snf5f/f_Arid1a_Cre_D2 (19,057,061)
Snf5f/f_Arid1a_GFP_D-3 (36,222,220)
Snf5f/f_Arid1a_GFP_D2 (39,307,130)
Snf5f/f_SS18_Cre_D-3 (22,418,225)
Snf5f/f_SS18_Cre_D2 (21,648,710)
Snf5f/f_SS18_GFP_D-3 (22,320,591)
Snf5f/f_SS18_GFP_D2 (16,099,864)
Snf5f/f_input_Cre_D-3 (47,433,445)
Snf5f/f_input_Cre_D2 (24,655,875)
Snf5f/f_input_GFP_D-3 (35,883,250)
Snf5f/f_input_GFP_D2 (38,864,958)
Snf5f/f_RNA_Cre_D-3 (12,877,211)
Snf5f/f_RNA_Cre_D2 (15,634,743)
Snf5f/f_RNA_GFP_D-3 (13,613,907)

Snf5f/f_RNA_GFP_D2 (12,959,464)
Snf5f/f_ATAC_Cre_D-3 (14,489,535)
Snf5f/f_ATAC_Cre_D2 (22,909,083)
Snf5f/f_ATAC_GFP_D-3 (18,885,501)
Snf5f/f_ATAC_GFP_D2 (20,853,649)
Mll3-/-Mll4f/f_CEBPb_Cre_Brg1_D-3 (16,451,535)
Mll3-/-Mll4f/f_CEBPb_GFP_Brg1_D-3 (24,025,378)
Mll3-/-Mll4f/f_Vec_Cre_Brg1_D-3 (17,080,750)
Mll3-/-Mll4f/f_Vec_GFP_Brg1_D-3 (24,779,208)
Mll3-/-Mll4f/f_CEBPb_Cre_input_D-3 (20,564,490)
Mll3-/-Mll4f/f_CEBPb_GFP_input_D-3 (18,314,028)
Mll3-/-Mll4f/f_Vec_Cre_input_D-3 (33,379,592)
Mll3-/-Mll4f/f_Vec_GFP_input_D-3 (25,913,157)
Mll3-/-Mll4f/f_CEBPb_Cre_Arid1a_D-3 (26,281,779)
Mll3-/-Mll4f/f_CEBPb_GFP_Arid1a_D-3 (27,482,397)
Mll3-/-Mll4f/f_Vec_Cre_Arid1a_D-3 (24,633,974)
Mll3-/-Mll4f/f_Vec_GFP_Arid1a_D-3 (27,428,402)
Mll3-/-Mll4f/f_CEBPb_Cre_ATAC_D-3 (15,421,763)
Mll3-/-Mll4f/f_CEBPb_GFP_ATAC_D-3 (27,051,879)
Mll3-/-Mll4f/f_Vec_Cre_ATAC_D-3 (20,213,657)
Mll3-/-Mll4f/f_Vec_GFP_ATAC_D-3 (25,959,886)
BPbrm1_RNA_Myf5Cre_D7 (36,005,366)
BPbrm1_RNA_flox_D7 (40,161,820)
Brg1AID/AID_Cebpb_Tir1_D-3_ATAC (20,260,222)
Brg1AID/AID_Cebpb_Vec_D-3_ATAC (25,253,145)
Brg1AID/AID_Vec_Tir1_D-3_ATAC (21,337,109)
Brg1AID/AID_Vec_Vec_D-3_ATAC (15,913,067)
Brg1AID/AID_Cebpb_Tir1_D-3_CEBPb (28,104,516)
Brg1AID/AID_Cebpb_Vec_D-3_CEBPb (27,609,637)
Brg1AID/AID_Vec_Tir1_D-3_CEBPb (25,771,879)
Brg1AID/AID_Vec_Vec_D-3_CEBPb (24,527,861)
Brg1AID/AID_Cebpb_Tir1_D-3_H3K27ac (25,139,573)
Brg1AID/AID_Cebpb_Vec_D-3_H3K27ac (31,207,683)
Brg1AID/AID_Vec_Tir1_D-3_H3K27ac (26,354,316)
Brg1AID/AID_Vec_Vec_D-3_H3K27ac (25,909,326)
Brg1AID/AID_Cebpb_Tir1_D-3_input (17,639,982)
Brg1AID/AID_Cebpb_Vec_D-3_input (15,923,916)
Brg1AID/AID_Vec_Tir1_D-3_input (17,699,402)
Brg1AID/AID_Vec_Vec_D-3_input (14,417,954)
Brg1AID/AID_Cebpb_Tir1_D-3_MLL4 (23,408,640)
Brg1AID/AID_Cebpb_Vec_D-3_MLL4 (26,284,930)
Brg1AID/AID_Vec_Tir1_D-3_MLL4 (31,135,287)
Brg1AID/AID_Vec_Vec_D-3_MLL4 (19,466,243)

Antibodies

Anti-ARID2/BAF200 (E-3, sc-166117X), nti-C/aEBPβ (C-19, sc-150X), anti-C/EBPα (144AA, sc-61X) and anti-PPARγ (H-100, sc-7196X) were from Santa Cruz. Anti-SMARCA4/BRG1 (ab110641) and anti-H3K27ac (ab4729) were from Abcam. Anti-ARID1A/BAF250A (D2A8U, #12354), anti-SS18 (D6I4Z, #21792) and anti-CBP (D6C5, #7389) were from Cell Signaling. Anti-BRD9 (#61537) was from Active Motif. Anti-H3K4me1 (13-0040) was from EpiCypher. Homemade anti-MLL4 antibody was used for ChIP-Seq.

Peak calling parameters

For ChIP-Seq of histone modifications (H3K4me1 and H3K27ac), the window size of 200 bp, the gap size of 200 bp, and the false discovery rate (FDR) threshold of 1E-3 were used. For ChIP-Seq of non-histone factors, the window size of 50 bp, the gap size of 50 bp, and the FDR threshold of 1E-10 were used. Number of called peak numbers are as follows:
Mll3-/-Mll4f/f_BAF200_GFP_D-3 (22,306)
Mll3-/-Mll4f/f_BAF200_GFP_D2 (5,469)
Mll3-/-Mll4f/f_SS18_Cre_D-3 (24,034)
Mll3-/-Mll4f/f_SS18_Cre_D2 (41,884)
Mll3-/-Mll4f/f_SS18_GFP_D-3 (68,479)
Mll3-/-Mll4f/f_SS18_GFP_D2 (17,301)
Mll3-/-Mll4f/f_Brg1_Cre_D-3 (66,215)
Mll3-/-Mll4f/f_Brg1_Cre_D2 (44,408)
Mll3-/-Mll4f/f_Brg1_GFP_D-3 (65,912)
Mll3-/-Mll4f/f_Brg1_GFP_D2 (61,708)
Snf5f/f_CEBPa_GFP_D2 (5,793)
Snf5f/f_CEBPb_GFP_D2 (64,353)
Snf5f/f_PPARg_GFP_D2 (7,197)
Snf5f/f_Brg1_Cre_D-3 (71,669)
Snf5f/f_Brg1_Cre_D2 (56,905)
Snf5f/f_Brg1_GFP_D-3 (82,386)
Snf5f/f_Brg1_GFP_D2 (47,304)
Snf5f/f_MLL4_Cre_D-3 (37,794)
Snf5f/f_MLL4_Cre_D2 (38,885)
Snf5f/f_MLL4_GFP_D-3 (38,281)
Snf5f/f_MLL4_GFP_D2 (61,473)
Snf5f/f_CBP_Cre_D-3 (18,545)
Snf5f/f_CBP_Cre_D2 (16,896)
Snf5f/f_CBP_GFP_D-3 (30,117)

Snf5f/f_CBP_GFP_D2 (15,950)
Snf5f/f_H3K4me1_Cre_D-3 (84,041)
Snf5f/f_H3K4me1_Cre_D2 (85,517)
Snf5f/f_H3K4me1_GFP_D-3 (85,716)
Snf5f/f_H3K4me1_GFP_D2 (83,262)
Snf5f/f_H3K27ac_Cre_D-3 (46,603)
Snf5f/f_H3K27ac_Cre_D2 (47,538)
Snf5f/f_H3K27ac_GFP_D-3 (45,017)
Snf5f/f_H3K27ac_GFP_D2 (46,153)
Snf5f/f_Brd9_GFP_D-3 (7,082)
Snf5f/f_Brd9_GFP_D2 (2,949)
Snf5f/f_Arid1a_Cre_D-3 (23,688)
Snf5f/f_Arid1a_Cre_D2 (18,294)
Snf5f/f_Arid1a_GFP_D-3 (45,627)
Snf5f/f_Arid1a_GFP_D2 (36,371)
Snf5f/f_SS18_Cre_D-3 (25,692)
Snf5f/f_SS18_Cre_D2 (20,423)
Snf5f/f_SS18_GFP_D-3 (43,559)
Snf5f/f_SS18_GFP_D2 (22,118)
Mll3-/-Mll4f/f_CEBPb_Cre_Brg1_D-3 (43,718)
Mll3-/-Mll4f/f_CEBPb_GFP_Brg1_D-3 (68,176)
Mll3-/-Mll4f/f_Vec_Cre_Brg1_D-3 (47,817)
Mll3-/-Mll4f/f_Vec_GFP_Brg1_D-3 (67,400)
Mll3-/-Mll4f/f_CEBPb_Cre_Arid1a_D-3 (52,349)
Mll3-/-Mll4f/f_CEBPb_GFP_Arid1a_D-3 (68,540)
Mll3-/-Mll4f/f_Vec_Cre_Arid1a_D-3 (53,161)
Mll3-/-Mll4f/f_Vec_GFP_Arid1a_D-3 (57,723)
Brg1AID/AID_Cebpb_Tir1_D-3_CEBPb (42,497)
Brg1AID/AID_Cebpb_Vec_D-3_CEBPb (61,615)
Brg1AID/AID_Vec_Tir1_D-3_CEBPb (27,076)
Brg1AID/AID_Vec_Vec_D-3_CEBPb (41,422)
Brg1AID/AID_Cebpb_Tir1_D-3_H3K27ac (44,090)
Brg1AID/AID_Cebpb_Vec_D-3_H3K27ac (50,869)
Brg1AID/AID_Vec_Tir1_D-3_H3K27ac (43,382)
Brg1AID/AID_Vec_Vec_D-3_H3K27ac (46,500)
Brg1AID/AID_Cebpb_Tir1_D-3_MLL4 (25,304)
Brg1AID/AID_Cebpb_Vec_D-3_MLL4 (20,112)
Brg1AID/AID_Vec_Tir1_D-3_MLL4 (43,760)
Brg1AID/AID_Vec_Vec_D-3_MLL4 (23,672)

Data quality

For ChIP-Seq of histone modifications (H3K4me1 and H3K27ac), peaks with the false discovery rate (FDR) < 1E-3 were included in the data analysis. For ChIP-Seq of non-histone factors, peaks with FDR < 1E-10 were used.

Software

Software for ChIP-Seq analysis include bowtie2, SICER algorithm.
