# Supplementary material for: Interplay of BAF and MLL4 promotes cell type-specific enhancer activation
Source: Nat Commun. 2021 Mar 12;12:1630. doi: 10.1038/s41467-021-21893-y (PMC7955098; doi:10.1038/s41467-021-21893-y)
Supplement: Supplementary file 4 — Description of Additional Supplementary Files [file 41467_2021_21893_MOESM4_ESM.pdf]

Descriptions of Additional Supplementary Files

Supplementary Data 1

Description: Raw data on MLL4 IP-mass spectrometry. Shown is a full list of MLL4-associated proteins in mouse embryonic stem cells identified by IP-Mass spectrometry.

Supplementary Data 2

Description: Raw data on UTX IP-mass spectrometry. Shown is a full list of UTX-associated proteins in mouse embryonic stem cells identified by IP-Mass spectrometry.
